# Description of a new species of the genus *Protracheoniscus* Verhoeff, 1917 and redescription of *Protracheoniscuskryszanovskii* Borutzky, 1957 from the southeast of European Russia (Isopoda, Oniscidea, Agnaridae)

**DOI:** 10.3897/zookeys.801.23167

**Published:** 2018-12-03

**Authors:** Konstantin B. Gongalsky, Ilya S. Turbanov, Dmitry A. Medvedev, Julia S. Volkova

**Affiliations:** 1 A.N. Severtsov Institute of Ecology and Evolution of Russian Academy of Sciences, Moscow, Russia A.N. Severtsov Institute of Ecology and Evolution of Russian Academy of Sciences Moscow Russia; 2 M.V. Lomonosov Moscow State University, Moscow, Russia M.V. Lomonosov Moscow State University Moscow Russia; 3 I.D. Papanin Institute of Biology of Inland Waters of Russian Academy of Sciences, Borok, Russia I.D. Papanin Institute of Biology of Inland Waters of Russian Academy of Sciences Borok Russia; 4 Cherepovets State University, Cherepovets, Russia Cherepovets State University Cherepovets Russia; 5 Ulyanovsk State University, Ulyanovsk, Russia Ulyanovsk State University Ulyanovsk Russia

**Keywords:** COI, *
Desertoniscus
*, Hungary, Isopoda, Kalmykia, new species, Oniscidea, *
Protracheoniscus
*, phylogenetic analysis, woodlouse

## Abstract

A new species of woodlice, *Protracheoniscuspokarzhevskii* Gongalsky & Turbanov, **sp. n.** is described from Kalmykia, NE Pre-Caspian region, Russia. *Protracheoniscuskryszanovskii* Borutzky, 1957 from the same area is also redescribed. Diagnostic features of these species as well as affinities within the genus are provided and discussed.

## Introduction

The highest diversity of terrestrial isopods in Russia is recorded in the Black Sea area and in the Caucasus, depending mostly on the availability of temperature sufficient to complete their life cycle and on appropriate soil moisture. Within the former USSR, these regions as well as the Central Asia are relatively well surveyed with many records of terrestrial isopods (Borutzky 1945, 1957, Eshaghi et al. 2015). However, there are gaps with very few isopod records across this territory, such as in the north of the Caspian region (Kuznetsova and Gongalsky 2012). The low Volga River basin attracts attention due to a unique position in Europe with natural steppes and sometimes with semi-deserts.

During a study of soil fauna in the surroundings of rice paddies in NE Kalmykia, a number of isopod species were recorded. Some of them were species new to Russia, and some have not been described yet. One new species of *Protracheoniscus* Verhoeff, 1917 is described. A species in the same genus described by EV Borutzky (1957) was collected as well. It has not been recorded for 60 years since description, and only sketch drawings of the diagnostic features of this species were available by now. A re-description of this species is provided below.

## Materials and methods

Sampling was done by the first author and his colleagues in the steppe ecosystems of Kalmykia in 2016–2017. Woodlice were collected by hand and fixed in 96% ethanol. Terminology used in the species description is mainly based on Vandel (1960). Processing and dissections were done by using a Leica MZ8 binocular microscope. Micro preparations of diagnostic body appendages were done in euparal (Carl Roth GmbH). Line drawings were executed with the help of an Olympus BX41 microscope supplied with an Olympus U-DA camera lucida. The material is deposited in the collection of the Zoological Museum of Moscow University, Russia (**ZMMU**), and partly retained in the private collection of the author (AN Severtsov Institute of Ecology and Evolution of Russian Academy of Sciences, Moscow, Russia), as indicated below.

*Phylogenetic analysis.* To confirm the validity of *P.pokarzhevskii* sp. n., a phylogenetic analysis was undertaken. In the molecular analysis, we used also specimens of *P.politus* from Budapest, Hungary, *P.pokarzhevskii* sp. n., *P.kryszanovskii*, *P.nogaicus*, *P.major* and *Desertoniscuszaitsevi* from Kalmykia, Russia. In the phylogenetic construction, a number of species from GenBank (Table 1) were used as outgroup taxa. To isolate total DNA, pereopods 4 or 5 were used from individuals fixed in 96% ethanol. Total cell DNA was isolated using a QIAamp DNA Investigator Kit (Qiagen, Germany). For the analysis of genetic variability, fragments of the mtDNA COI loci were used. The study was performed using full-length fragments obtained by amplification of mtDNA with primers HCO2198 / LCO1490 (Folmer et al. 1994).

**Table 1.** List of specimens, sampling sites and accession numbers of the sequences for COI mt DNA included in this study. References are given for sequences obtained from GenBank.

Specimen	Locality	Reference / or year of sampling	
		COI mtDNA	Reference
*Desertoniscuszaitsevi* Gongalsky, 2017	Russia, Republic of Kalmykia, 1 km N of Bolshoy Tsaryn (47.9040N, 45.3929E) 29.04.2017, K. Gongalsky leg.	MH400725	This study / 2018
*Protracheoniscuskryszanovskii* Borutzky, 1957	Russia, Republic of Kalmykia, 1 km W of Tsagan Nur, bank of lake Sarpa (47.362N, 45.201E), 27.04.2017 K. Gongalsky leg.	MH400727	This study / 2018
*Protracheoniscusmajor* (Dollfus, 1903)	Russia, Republic of Kalmykia, 1 km N of Bolshoy Tsaryn (47.9040N, 45.3929E) 29.04.2017, K. Gongalsky leg.	MH400726	This study / 2018
*Protracheoniscusnogaicus* Demianowicz, 1932	Russia, Republic of Kalmykia, 1 km N of Bolshoy Tsaryn (47.9040N, 45.3929E) 29.04.2017, K. Gongalsky leg.	MH400724	This study / 2018
*Protracheoniscuspokarzhevskii* sp. n.	Russia, Republic of Kalmykia, 1 km N of Bolshoy Tsaryn, (47.9040N, 45.3929E) 29.04.2017, K. Gongalsky leg.	MG696253, MH400723	This study / 2017–18
*Protracheoniscuspolitus* (C. Koch, 1841)	Hungary, Budapest, Janos Hegy Mt. (47.5158N, 18.9602E) 29.08.2017, K. Gongalsky leg.	MG696252	This study / 2017
*Burmoniscuskathmandius* (Schmalfuss, 1983)	Nepal	LC075192	Karasawa 2016
*Ligiabaudiniana* Milne-Edwards, 1840	Colombia	KF555872	Santamaria et al. 2014

The polymerase chain reaction was carried out on a Bio-Rad T 100 thermocycler (Bio-Rad, USA) in a specially selected temperature regime: the initial denudation of 95 °C was 5 min; annealing of 95 °C for 35 seconds, 48 °C for 40 seconds, 72 °C for 40 seconds (35 cycles), the final elongation of 72 °C lasted for 7 minutes. For the PCR, a set of reagents for the amplification of “5× Mas Mix-2025” manufactured by Dialat Ltd (Moscow, Russia) was used. The 15 μl reaction mixture contained 1 μl total DNA, 3 μl mix and 1 μl of each primer. The amplification products were separated by electrophoresis in 1.5% agarose gel in 1× TBE and visualized with ethidium bromide. The DNA sequence was determined with a forward and reverse primer using the Big Dye 3.1 kits on an ABI 3500 genetic analyzer from Applied Biosystems, USA, in a POP7 polymer environment.

The obtained sequences were aligned with the help of BioEdit v. 5.0.9 software. The obtained fragments of sections of COI mtDNA genes were used in phylogenetic analysis. The phylogenetic tree was built in MEGA 6.0.

In the species assessment, the Neighbor Joining (NJ) method was used, based on all the sequences obtained and with the calculation of bootstrap support of branch nodes (1000 replicates). The loci of COI mtDNA *Burmoniscuskathmandius* (Karasawa, 2016) and *Ligiabaudiniana* (Santamaria et al., 2014) were used as outgroup taxa. The results obtained are presented in the form of a phylogenetic tree (Figure 11).

## Taxonomy

### Class Malacostraca Latreille, 1802

#### Order Isopoda Latreille, 1817

##### Family Agnaridae Schmidt, 2003

###### Genus *Protracheoniscus* Verhoeff, 1917

####### Protracheoniscus pokarzhevskii

Taxon classificationAnimaliaIsopodaAgnaridae

Gongalsky & Turbanov sp. n.

http://zoobank.org/C2AC4DE5-A416-4637-8AF7-47B8E25601C5

######## Type material.

**Holotype**: ♂ (ZMMU), Russia, Republic of Kalmykia, 1 km N of Bolshoy Tsaryn (47.9040N, 45.3929E), dry steppes (*Artemisiaaustriaca*, *Festucavalesiaca*, *Tanacetumachilleifolium*), 29.04.2017, K. Gongalsky leg. **Paratypes**: 2 ♂♂, 2 ♀♀ (ZMMU), 3 ♂♂, 4 ♀♀ (private collection of K. Gongalsky), same date, location and collector.

######## Other material examined.

*Protracheoniscuspolitus* (C. Koch, 1841): 3 ♂♂, 6 ♀♀, Hungary, Budapest, János-Hegy (47.5158N, 18.9602E), 29.08.2017, K. Gongalsky leg. *Protracheoniscusnogaicus* Demianowicz, 1932: 2 ♂♂, Russia, Republic of Kalmykia, 1 km N of Bolshoy Tsaryn (47.9040N, 45.3929E), 29.04.2017, K. Gongalsky leg. *Protracheoniscusmajor* (Dollfus, 1903): 6 ♂♂, 15 ♀♀, same date, location, and collector. *Desertoniscuszaitsevi* Gongalsky, 2017: 3 ♂♂, 5 ♀♀, same date, location, and collector.

######## Diagnosis.

A species of *Protracheoniscus* characterized by the antennal flagellum with the articles of a ratio close to 1:1; male exopod of pleopod 1 with almost rounded apex; telson with distal part elongated and distal corner forming triangle; and one of four medial spines of outer endite of maxillula is twice as small as the others.

######## Description.

**Figure 1.**
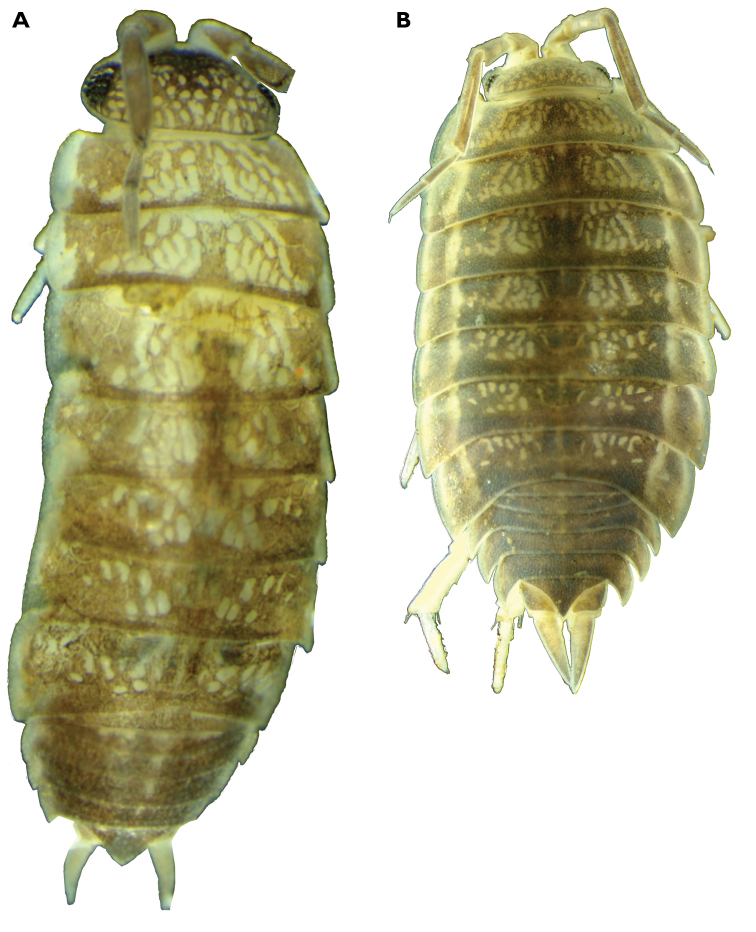
Dorsal view of male paratype of *Protracheoniscuspokarzhevskii* sp. n., 4 mm (**A**), and male *Protracheoniscuskryszanovskii*Borutzky 1957, 9 mm (**B**), from Kalmykia, SE of European Russia.

**Figure 2.**
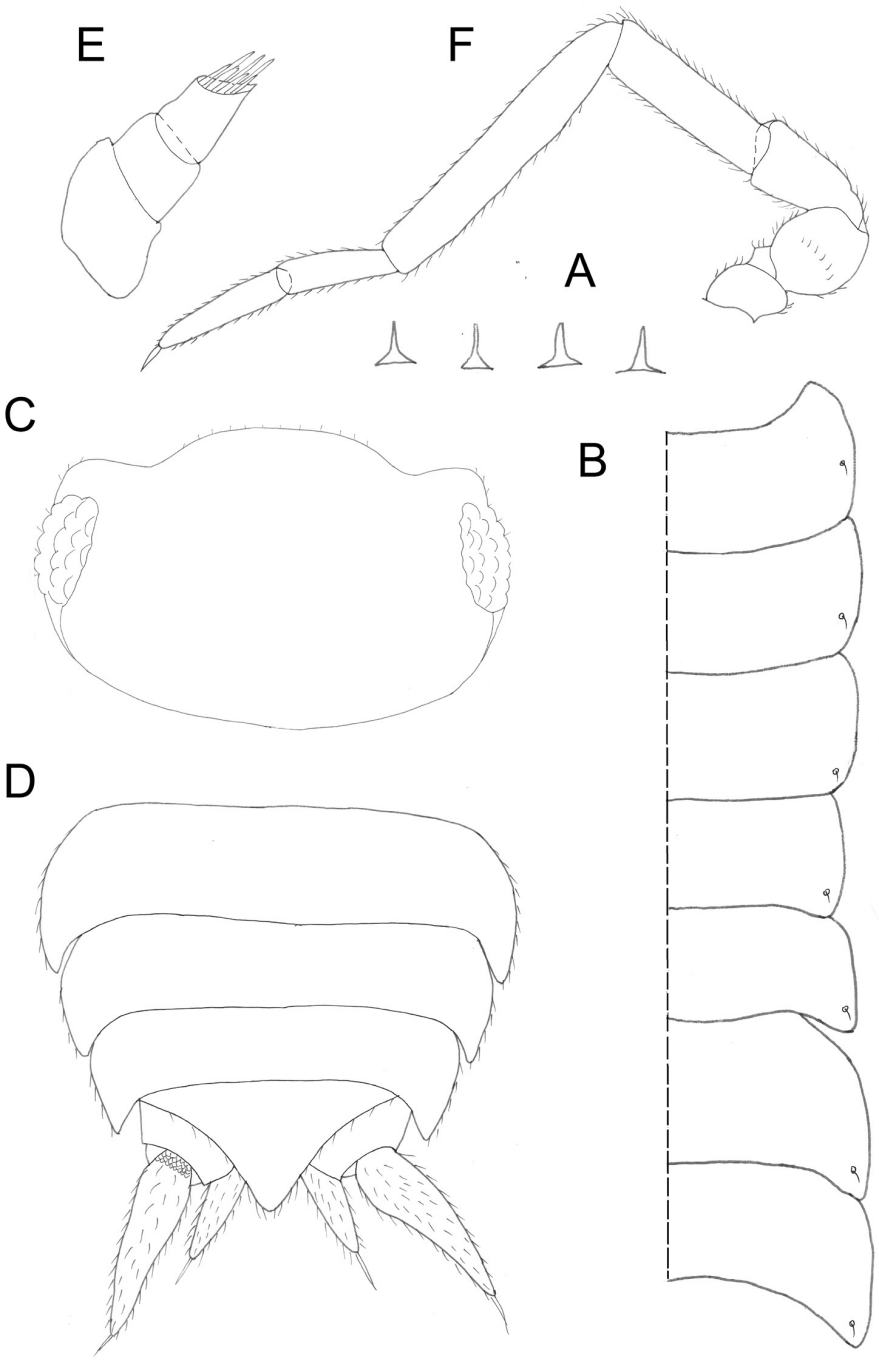
*Protracheoniscuspokarzhevskii* sp. n.: **A** dorsal scale-setae **B** pereon edge **C** head **D** pleonites 3–5, telson and uropods **E** antennula **F** antenna (female, paratype).

*Somatic characters.* Maximum body length: male 5.0 mm; female 5.5 mm. Holotype body length 4.7 mm. Body color dark grey-brown; frontal part of head much darker than rest of the body; light grey-brown spots at base of coxal plates of pereonal segments 2–7 (Figure 1A). Dorsal surface of tergites smooth. Posterior edges of coxal plates of pereonites straight (Figs 1A, 2B). Distal part of head covered with scattered sharp triangular dorsal setae (Figure 2A). Noduli laterales on pereonites located close to coxal plates edges (Figure 2B). Body relatively elongated; pleon not continuous with pereon outline (Figure 1A). Cephalic lobes poorly developed; distal edge of median lobe rounded (Figure 2C). Telson with distal part elongated and distal corner forming triangle (Figure 2D).

**Figure 3.**
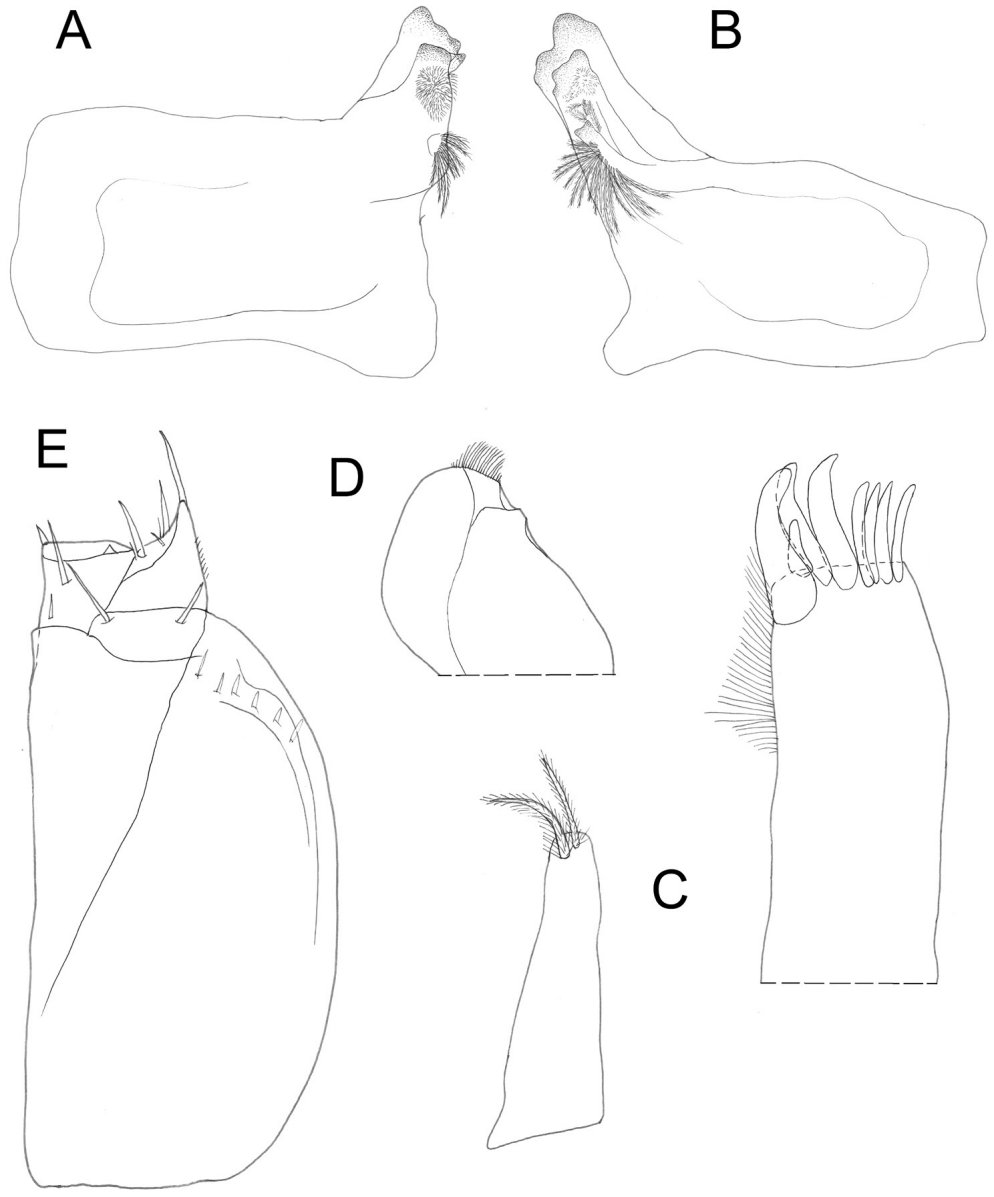
*Protracheoniscuspokarzhevskii* sp. n.: **A** left mandible **B** right mandible **C** maxillula **D** maxilla **E** maxilliped (female, paratype).

*Appendages.* Uropods (Figure 2D) colored as dorsal body surface; exopods elongated. Antennula of three articles (Figure 2E); first article wide and relatively long, second article slightly shorter than first, third article almost as long as first and narrow, bearing a tuft of aesthetascs at apex. Antenna reaching pereonite 3 (Figure 1A); flagellum of two articles, proximal article slightly shorter than distal one (Figure 2F). Left mandible (Figure 3A); pars incisiva with two teeth and lacinia mobilis with straight edge; molar penicil consisting of ca. ten setae. Right mandible smaller than left mandible, pars incisiva with three teeth and lacinia mobilis with two teeth, molar penicil consisting of ca. ten setae (Figure 3B). Maxillula (Figure 3C): medial corner of inner endite with two strong penicils; apical edge of outer endite bearing 4 + 4 teeth with simple tips, with one tooth in medial group twice smaller than other three. Maxilla with bilobate edge, medial half of apical edge of outer lobe with dense brush of short setae (Figure 3D); inner margin with subapical tubercle. Maxilliped with outer corner of endite with two acute tips and large spine near inner corner (Figure 3E). Pleopods (Figure 5). All exopods with monospiracular covered lungs.

**Figure 4.**
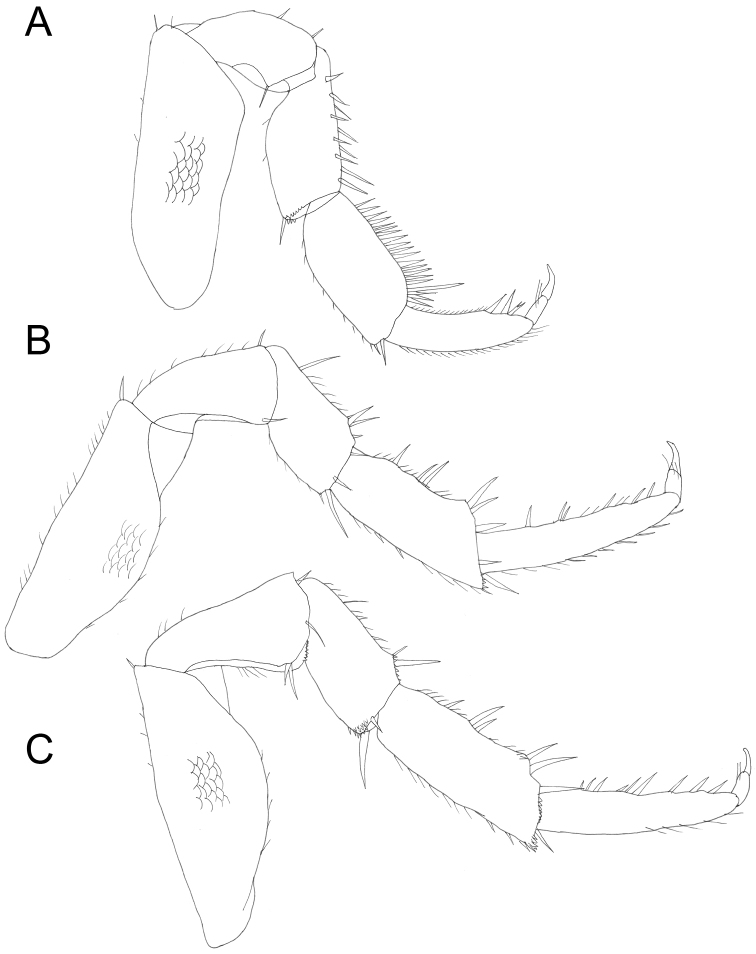
*Protracheoniscuspokarzhevskii* sp. n.: **A** pereopod 1 **B** pereopod 6 **C** pereopod 7 (male, paratype).

**Figure 5.**
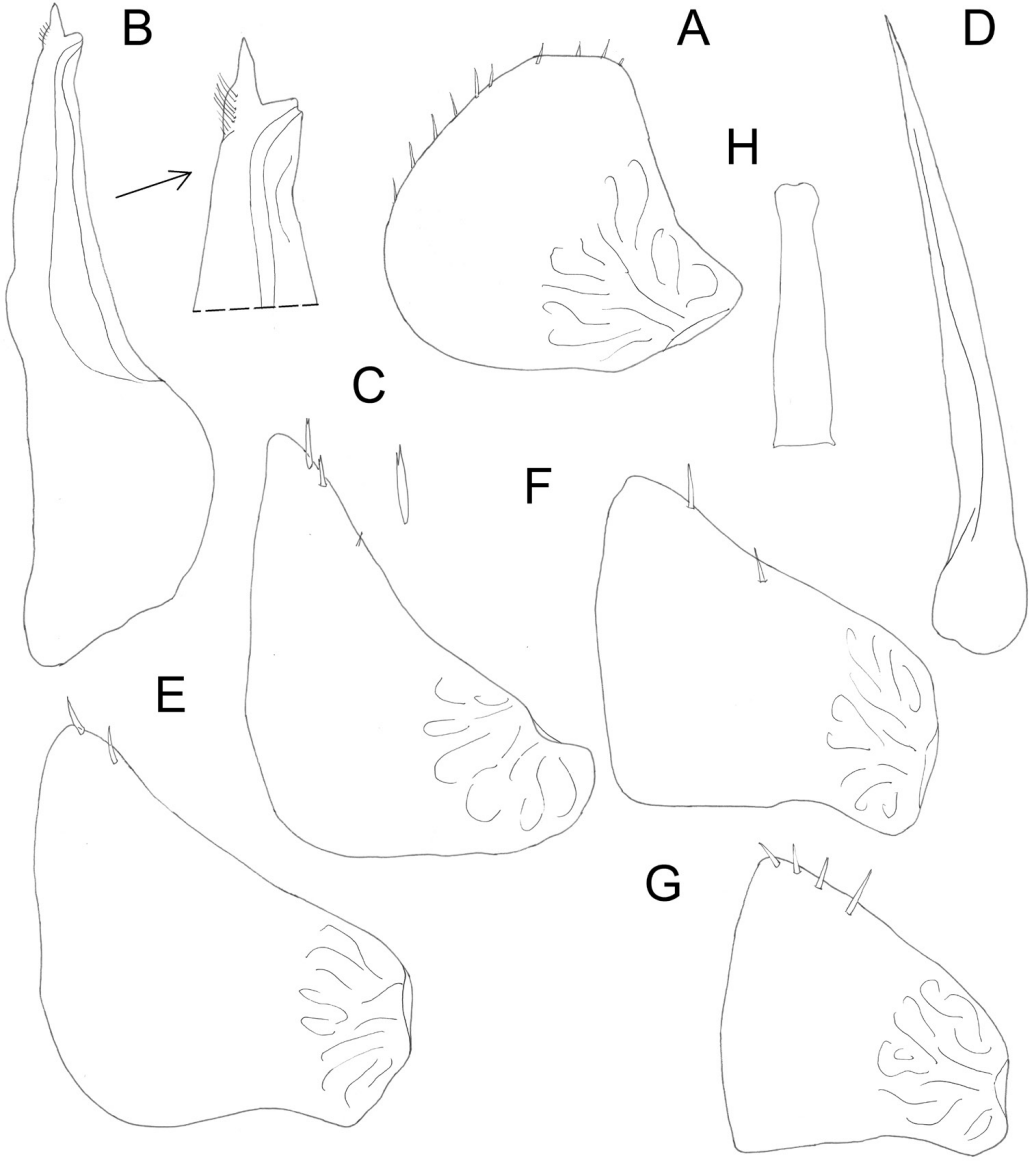
*Protracheoniscuspokarzhevskii* sp. n.: **A** exopod of pleopod 1 **B** endopod of pleopod 1 **C** exopod of pleopod 2 **D** endopod of pleopod 2 **E** exopod of pleopod 3 **F** exopod of pleopod 4 **G** exopod of pleopod 5 **H** genital papilla (male, paratype).

Male: Pereopods (Figure 4A–C): pereopod 1 with dactylus slightly bent ventrally; carpus with brush of setae with split tips. Pereopod 6 and 7 ischia with sternal margin straight. Genital papilla slightly extended at tip (Figure 5H). Exopod of pleopod 1 (Figure 5A) with almost rounded tip and ca. ten setae at apex, outer margin slightly concave. Endopod of pleopod 1 with split distal part: straight sharp triangular tip bearing row of spines and lateral bulb (Figure 5B). Pleopod 2: exopod triangular with concave outer margin bearing two setae with split tips (Figure 5C); endopod much longer than exopod, narrow, with parallel sides (Figure 5D). Pleopod 3–5: exopods (Figure 3E–F) trapezoidal, slightly decreasing in size from 3 to 5. Pleopod 5 exopod with sharp medial corner (Figure 5G).

######## Remarks.

This species is morphologically closest to *Protracheoniscuspolitus* (C Koch, 1841) in the similar shape of the endopodite of the male pleopod 1. However, the new species differs from *P.politus* in the following: (i) exopod of the male pleopod 1 with shorter posterior lobe; (ii) tip of endopod of pleopod 1 not bent laterally; (iii) telson with less concave sides and shorter and less acute tip; (iii) ratio of articles of flagellum is close to 1:1 (almost close to 1:2 in *P.politus*) (Gruner 1966; Tomescu et al. 2016).

Recently, two close species of *Protracheoniscus* were described from north Iran (Kashani and Hamidnia 2016). *Protracheoniscuspokarzhevskii* sp. n. differs from *P.kiabii* Kashani & Hamidnia, 2016 in (i) the noduli laterales located much closer to the pereonites’ margins; (ii) much shorter uropods; (iii) straight instead of concave sternal margin of the male pereopod 7 ischium; (iv) sharp tip of endopod of the male pleopod 1. From *P.golestanicus* Kashani & Hamidnia, 2016 it differs in (i) different shape of both exopod and endopod of the male pleopod 1; (ii) different position of noduli laterales. Evidently, a molecular analysis is needed for the complex of these small species of *Protracheoniscus*.

######## Distribution.

The new species has only been found between the Volga and Vostochnyi Manych Rivers. It occupies the steppes of Kalmykia (*Artemisiaaustriaca*, *Festucavalesiaca*, *Tanacetumachilleifolium*).

######## Etymology.

The species is named after Prof Dr Andrey D Pokarzhevskii (1946–2006), a prominent Russian soil zoologist who encouraged the first author to study terrestrial isopods.

####### Protracheoniscus kryszanovskii

Taxon classificationAnimaliaIsopodaAgnaridae

Borutzky, 1957

######## Material examined.

1 ♂, 1 ♀, 6 juveniles (ZMMU Mc-627); [USSR, Stavropol Region, bank of lake] Manych, 4 km NE of Divnoe, 20.05.1950. O. Kryzhanovsky leg. 2 ♂♂, 5 ♀♀ (ZMMU); Russia, Republic of Kalmykia, 1 km W of Tsagan Nur, bank of lake Sarpa. 27.04.2017 (47.362, 45.201), K. Gongalsky leg. 4 ♂♂, 8 ♀♀ (private collection of K. Gongalsky), same date and location.

######## Diagnosis.

*Protracheoniscuskryszanovskii* is characterized by the shape of cephalic lobes; exopod of male pleopod 1; and dactylus of male pereopods 6 and 7 widened in the middle (Borutzky 1957).

######## Re-description.

**Figure 6.**
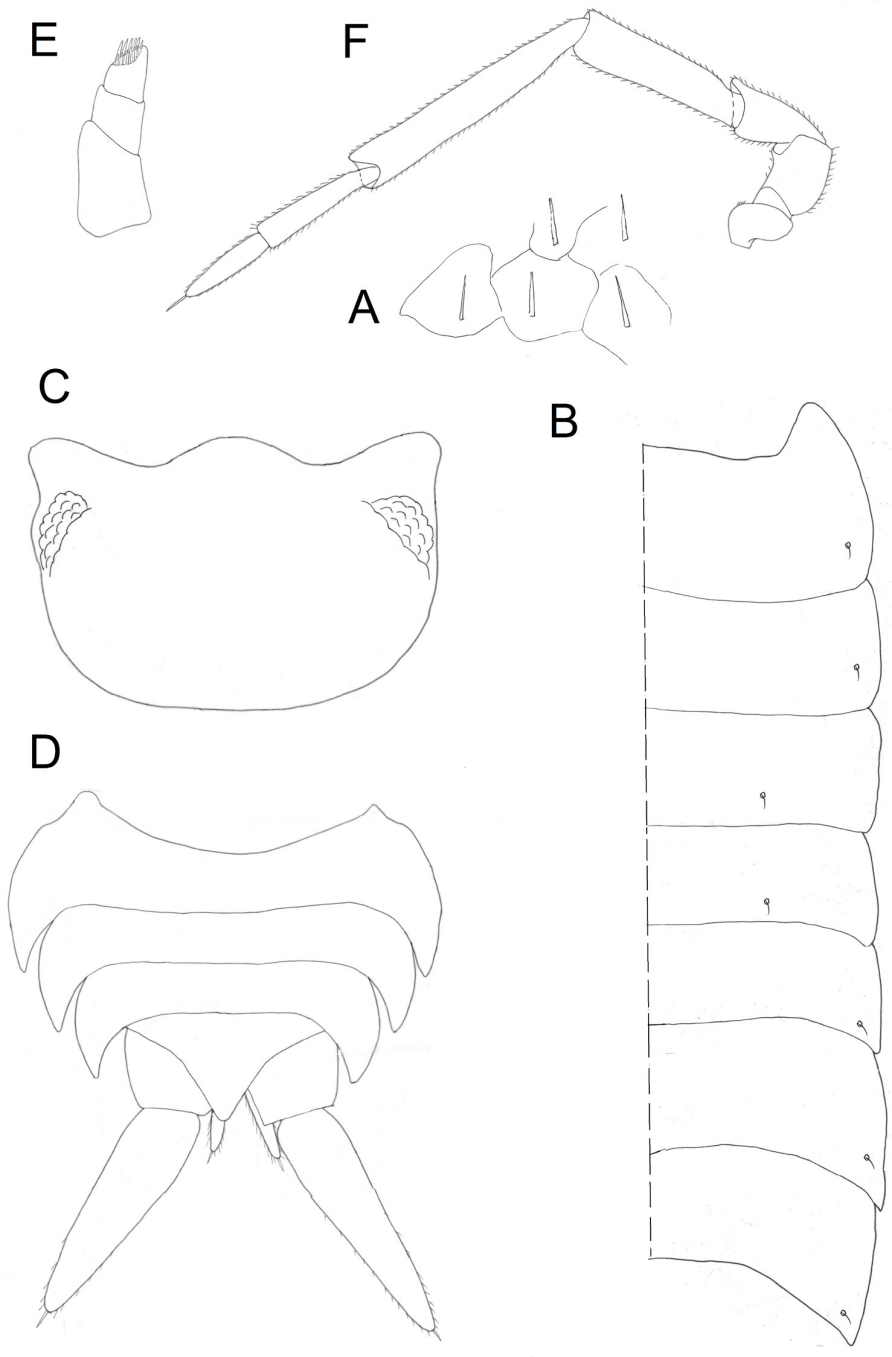
*Protracheoniscuskryszanovskii* Borutzky, 1957: **A** dorsal scale-setae **B** pereon edge **C** head **D** telson **E** antennula **F** antenna (female).

*Somatic characters.* Maximum body length: male 21 mm; female 14 mm (Borutzky 1957). Body color dark grey-brown white or yellow spots at base of coxal plates of pereonites 2–7(Figure 1B). Dorsal surface of tergites smooth. Posterior edges of coxal plates of pereonites straight (Figure 6B). Distal part of head covered with scattered sharp triangular dorsal scale-setae (Figure 6A). Noduli laterales on pereonites 3–4 located distinctly more distant from coxal plates lateral edges (Figure 6B). Body relatively elongated; pleon outline not continuous with pereon (Figure 1B). Cephalic lobes well developed; median lobe rounded (Figure 6C). Telson with distal part elongated and distal corner forming sharp triangle (Figure 6D).

**Figure 7.**
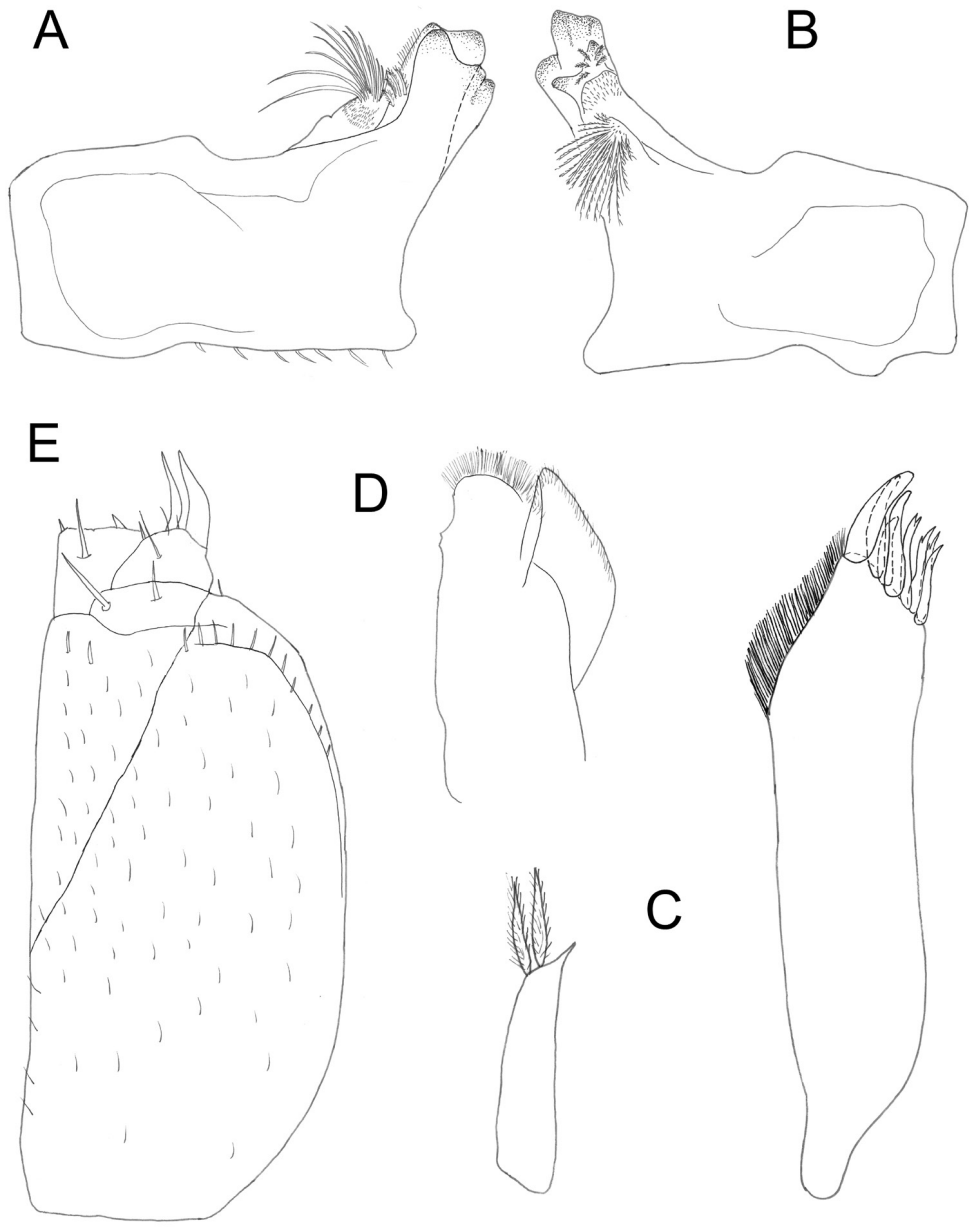
*Protracheoniscuskryszanovskii* Borutzky, 1957: **A** left mandible **B** right mandible **C** maxillula **D** maxilla **E** maxilliped (female).

*Appendages.* Uropods (Figure 6D) colored as dorsal surface of body; exopods elongated, widened in the middle. Telson not reaching endopods of uropods. Antennula with three articles (Figure 6E); first article wide and relatively long; second article 1.5–2 times shorter than first; third article almost as long as second and narrow, bearing a tuft of setae at apex. Antenna long, reaching pereonite 3 (Figure 1B); flagellum with proximal article 1.5 times longer than distal one (Figure 6F). Left mandible (Figure 7A) with pars incisiva with two teeth and lacinia mobilis with straight edge; molar penicil with ca. 12–15 setae. Right mandible smaller than left with pars incisiva with three teeth and lacinia mobilis with two teeth bearing five penicil setae; molar penicil with 15–18 setae (Figure 7B). Maxillula (Figure 7C): medial corner of inner endite with two strong penicils and sharp tip; apical edge of outer endite with 4 + 4 teeth, four of which apically cleft. Maxilla with bilobate edge, medial half of apical edge of inner lobe with a dense brush of short setae (Figure 7D); inner margin with subapical tubercle. Maxilliped with outer corner of endite with two acute tips and large spine near the inner corner (Figure 7E). All exopods of pleopods with monospiracular covered lungs.

**Figure 8.**
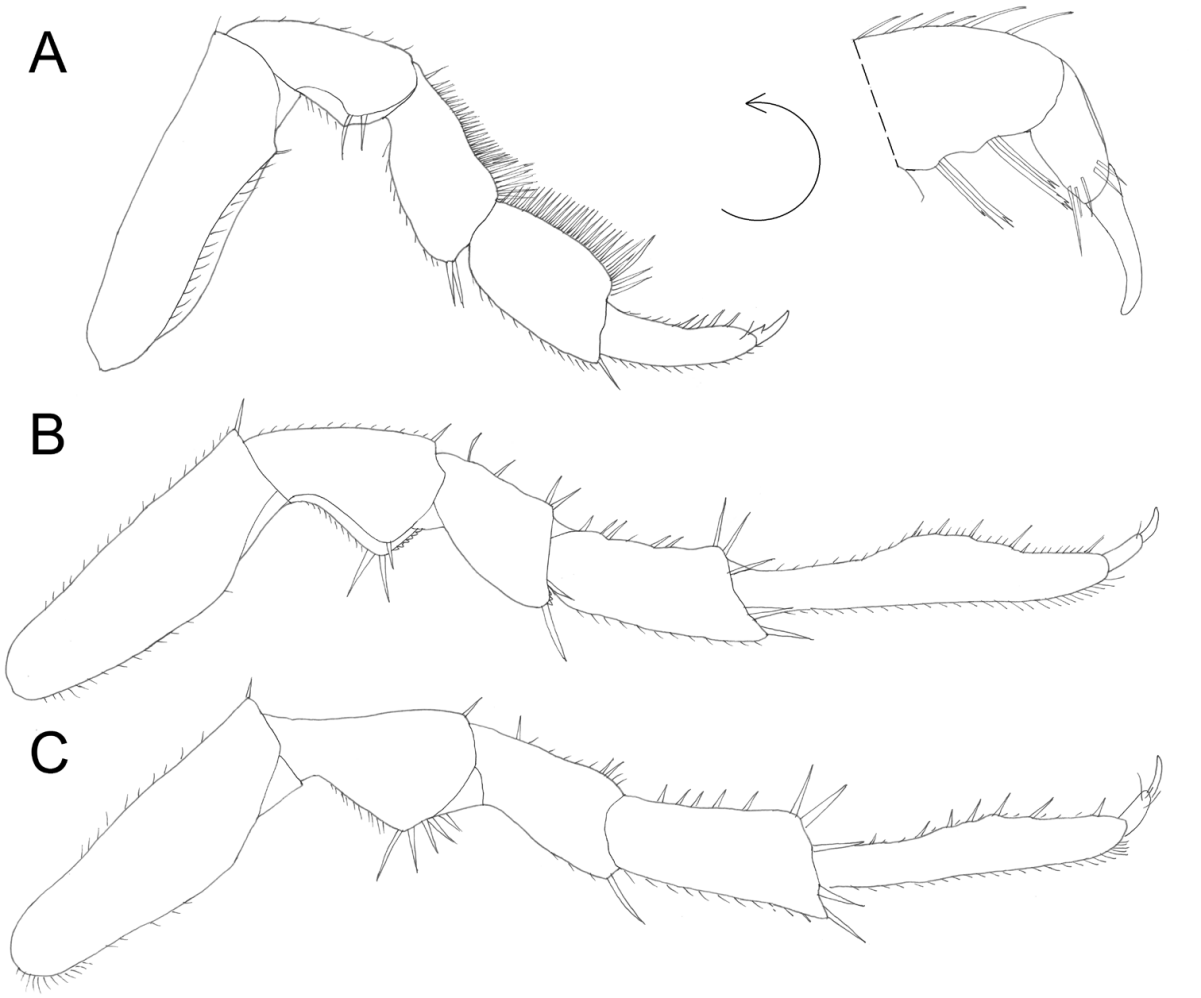
*Protracheoniscuskryszanovskii* Borutzky, 1957: **A** pereopod 1 **B** pereopod 6 **C** pereopod 7 (male).

**Figure 9.**
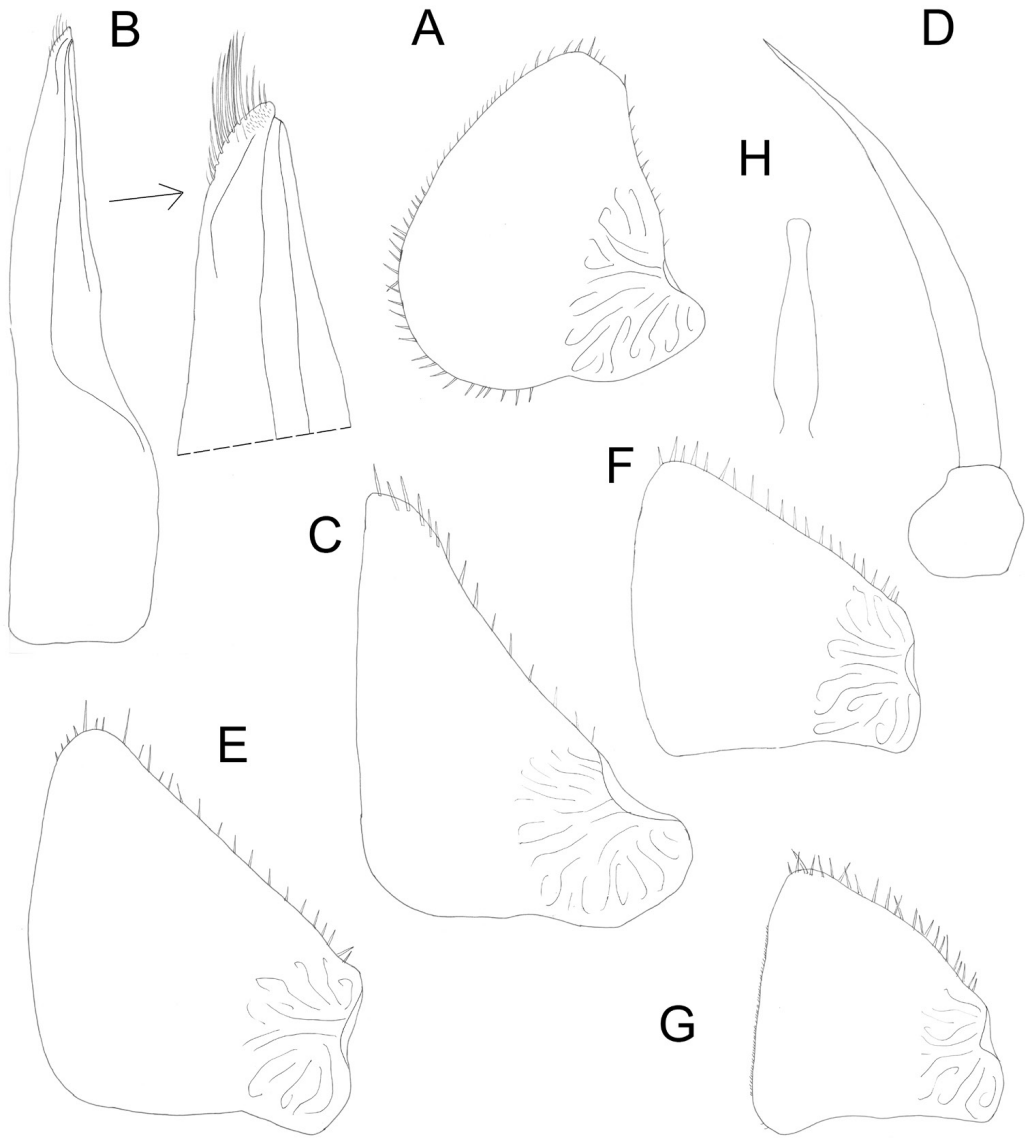
*Protracheoniscuskryszanovskii* Borutzky, 1957: **A** exopod of pleopod 1 **B** endopod of pleopod 1 **C** exopod of pleopod 2 **D** endopod of pleopod 2 **E** exopod of pleopod 3 **F** exopod of pleopod 4 **G** exopod of pleopod 5 **H** genital papilla (male).

Male: Pereopods (Figure 8A–C): Pereopod 1 (Figure 8A) with a brush of setae with split tips on merus and carpus; dactylus of pereopods 6 and 7 widened in the middle (Figure 8C). Genital papilla slightly inflated at tip (Figure 8H). Exopod of pleopod 1 (Figure 9A) with almost rounded tip and numerous small setae at apex, outer margin slightly concave with minute setae; endopod of pleopod 1 with triangular apical part with tuft of long setae on inner margin (Figure 9B). Pleopod 2: exopod triangular with straight outer margin bearing more than 15 setae (Figure 9C); endopod much longer than exopod, narrow, with parallel sides (Figure 9D). Pleopod 3–4 exopods (Figure 9E–F) trapezoidal, slightly decreasing in size. Pleopod 5 exopod triangular, with sharply rounded corners (Figure 9G).

######## Remarks.

This species belongs to the central Asian group of *Protracheoniscus* characterized by the position of noduli laterales on pereonites 3 and 4 at a distance from the lateral edge (Borutzky, 1957). The distinctive feature of the genus is the male dactylus of pereopod 6 and 7 widened in the middle. This species is the morphologically closest to *P.major* (Dollfus, 1903), from which it differs in lacking the enlargement of dactyli of male pereopods 6 and 7 (see Gruner 1966; Tomescu et al. 2016). *Protracheoniscusmajor* is one of the dominant woodlice species in Kalmykia and broadly distributed around the Caspian Sea (Kashani and Hamidnia 2016). The study of several specimens from the type locality confirmed the identity of our specimens with the type series designated by Borutzky (1957). Both the old collection and recent one of *P.kryszanovskii* showed substantial variability in the endopodite of the male pleopod 1.

######## Distribution.

**Figure 10.**
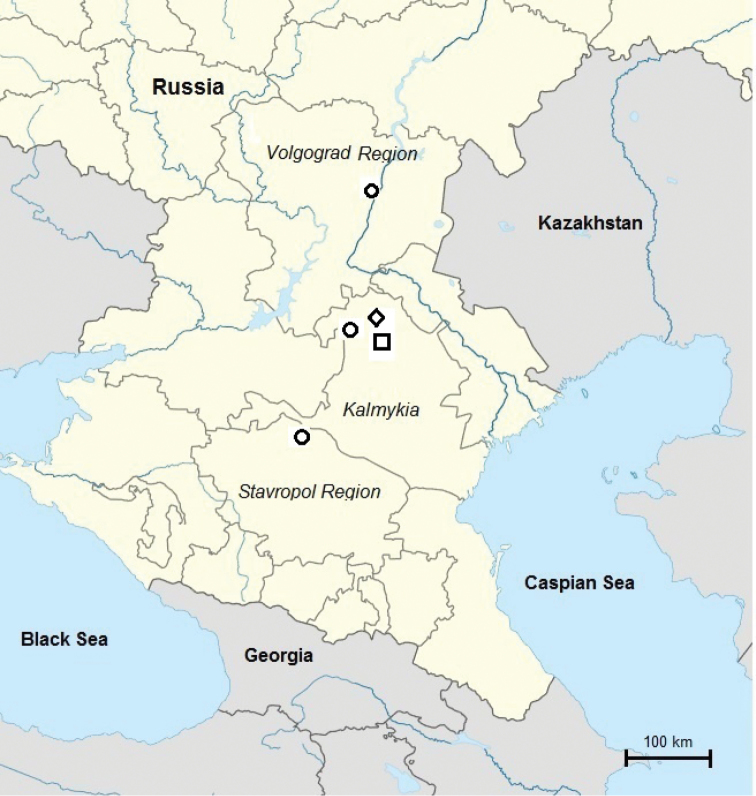
Distribution in the SE of European Russia of *Protracheoniscuskryszanovskii* from the original description by Borutzky (1957) (circles) and new location on Sarpa Lake, Kalmykia (square), and location of *Protracheoniscuspokarzhevskii* sp. n. in Bolshoi Tsaryn (diamond).

The species has been found between the Volga and Vostochnyi Manych Rivers so far (Figure 10). It occupies steppes of Kalmykia (*Artemisiaaustriaca*, *Festucavalesiaca*, *Tanacetumachilleifolium*) and is common in various biotopes being one of the dominant species in the region. The species prefers salted soils, namely the banks of lakes with salted water. In the surroundings of Kamyshin (Volgograd Region) it was found in the burrows of *Spermophilus* sp. and in leaf litter (Borutzky 1957).

######## Phylogenetic analysis.

The results of the pairwise distance analysis based on the analysis of the mtDNA COI gene (Table 2) show that the difference between *P.politus* and *P.pokarzhevskii* sp. n. by mtDNA COI is 23.0%. The differences between *P.pokarzhevskii* sp. n. from the types of outgroup taxa by mtDNA COI range from 24.0% to 30.0%. The results obtained indicate the species’ independence of *P.pokarzhevskii* sp. n. and a considerable divergence from the morphologically close *P.politus*.

**Figure 11.**
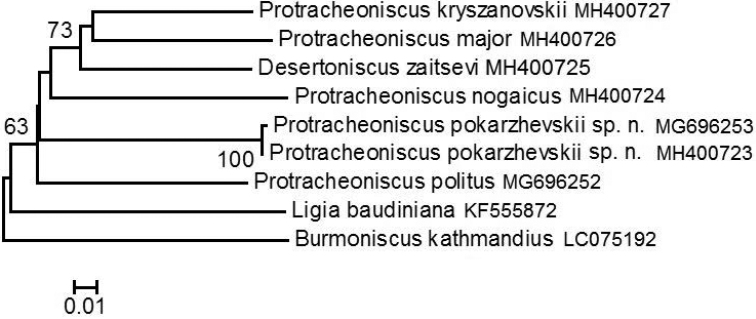
Phylogeny of six species of the genera *Protracheoniscus* and *Desertoniscus* with outgroup taxa based on the analysis of the mtDNA COI gene by the Neighbor Joining method (NJ) with the calculation of bootstrap support of branch sites (1000 replications).

**Table 2.** Estimates of pairwise sequence divergence (uncorrected p-distances) of gene COI mtDNA haplotypes.

		1	2	3	4	5	6	7	8	9
1	* Protracheoniscus kryszanovskii * MH400727	–								
2	* Protracheoniscus major * MH400726	0.15	–							
3	* Protracheoniscus nogaicus * MH400724	0.21	0.19	–						
4	* Protracheoniscus politus * MG696252	0.19	0.21	0.20	–					
5	*Protracheoniscuspokarzhevskii* sp.n. MG696253	0.20	0.21	0.21	0.20	–				
6	*Protracheoniscuspokarzhevskii* sp.n. MH400723	0.20	0.21	0.21	0.19	0.00	–			
7	* Desertoniscus zaitsevi * MH400725	0.16	0.16	0.20	0.19	0.19	0.19	–		
8	* Burmoniscus kathmandius * LC075192	0.23	0.25	0.25	0.23	0.24	0.24	0.24	–	
9	* Ligia baudiniana * KF555872	0.21	0.24	0.25	0.24	0.24	0.24	0.23	0.25	–

## Supplementary Material

XML Treatment for Protracheoniscus pokarzhevskii

XML Treatment for Protracheoniscus kryszanovskii
